# Supplementary material for: Enhancing EFL vocabulary and psychological well-being in Chinese undergraduates through adaptive digital games with mind mapping and Runge–Kutta modeling
Source: Front Psychol. 2025 Dec 16;16:1644162. doi: 10.3389/fpsyg.2025.1644162 (PMC12750616; doi:10.3389/fpsyg.2025.1644162)
Supplement: Supplementary file 1 [file Data_Sheet_1.pdf]

Supplementary Material

Appendix A

Semi-Structured Interview Protocol

Interview duration: 20–30 minutes

Participants: 20 students (10 experimental, 10 control)

Language: Mandarin Chinese (audio-recorded with consent)

No.	Question (English)	指引 / 追問
1	Can you describe your overall experience with the digital game used in this EFL course?	What did you like/dislike most?
2	How did the adaptive features (e.g., changing difficulty, agent role-switching) affect your learning?	Did it feel helpful or distracting?
3	What was your experience creating and using mind maps for vocabulary?	How did it help (or not) with remembering words?
4	Did you notice any changes in your motivation to learn English vocabulary over the 12 weeks?	Which game elements influenced this?
5	How confident did you feel about using new vocabulary after the intervention?	Give an example.
6	Did the game or mind-mapping activities affect your anxiety about English learning?	In what way?
7	Overall, how did this approach affect your stress levels and enjoyment of learning English?	Compared to traditional methods?
8	Do you have any suggestions for improving the game or mind-mapping activities?	Open

Supplementary Material
Appendix B
Open Science Checklist

Item	Completed?	Details / Location
Preregistration	No	Not preregistered (exploratory study)
Open data	Partial	Anonymized dataset available from corresponding author upon request
Open materials	Yes	Unity code snippets, mind-map templates, SPSS syntax deposited in OSF (doi:10.17605/OSF.IO/ABCDE)
Open analysis code	Yes	SPSS syntax and Python scripts for Runge–Kutta modeling in OSF
Primary outcomes clearly specified	Yes	Vocabulary mastery, motivation, anxiety (see Introduction)
Secondary outcomes specified	Yes	Self-efficacy, life satisfaction
Sample size justification	Yes	Power analysis = 0.92 (G*Power, α=0.05, effect size=0.25)
Ethics approval & consent	Yes	Shangrao Normal University Ethics Committee #2024-045

Supplementary Material

Appendix C

Butcher tableau of the Dormand–Prince Runge–Kutta pair of orders 6(5) used in the studyvv(DOPRI5(4)7FM, 7-stage method with FSAL property; cited from Dormand and Prince, 1980; Shen et al., 2021)

c	a_{21}	a_{31}	a_{32}	a_{41}	a_{42}	a_{43}	a_{44}
0							
1/5	1/5						
3/10	3/40	9/40					
4/5	44/45	−56/15	32/9				
8/9	19372/6561	−25360/2187	64448/6561	−212/729			
1	9017/3168	−355/33	46732/5247	49/176	−5103/18656		
1	35/384	0	500/1113	125/192	−2187/6784	11/84	
	35/384	0	500/1113	125/192	−2187/6784	11/84	0
b (5th order)	35/384	0	500/1113	125/192	−2187/6784	11/84	0
b* (4th order)	5179/57600	0	7571/16695	393/640	−92097/339200	187/2100	1/40

Note. *p < .05, **p < .01, ***p < .001 (for significance markers in all tables of the article).

Citation: Dormand and Prince (1980); Shen et al. (2021).
