# Supplementary material for: Enhancing EFL vocabulary and psychological well-being in Chinese undergraduates through adaptive digital games with mind mapping and Runge–Kutta modeling
Source: Front Psychol. 2025 Dec 16;16:1644162. doi: 10.3389/fpsyg.2025.1644162 (PMC12750616; doi:10.3389/fpsyg.2025.1644162)
Supplement: Supplementary file 2 [file Supplementary_file_1.docx]

**Appendix**

**A. Runge-Kutta Model Implementation**

- 1. Model Equations

The Runge-Kutta Pairs of Orders 6(5) were implemented using a Mathematica package adapted from Shen et al. (2021). The ODEs for vocabulary retention (𝑉) and anxiety (𝐴) were solved with adaptive step-size control. The numerical solution for anxiety is:

$A_{n+1}=A_{n}+h\sum_{i=1}^{9} b_{i}f_{i}$

Where$g_{i}=k_{3}\cdot S\left( t_{n}+c_{i}h \right)-k_{4}\cdot A_{n} ,$and$\hat{b}_{i}$ are coefficients for the fifth-order estimate, ensuring error control (Lin & Hwang, 2024).

- 1. Parameter Estimation

Rate constants $k_{1}=0.05, k_{2}=0.02, k_{3}=0.03, k_{4}=0.0$ were estimated using differential evolution, achieving a 95% fit for vocabulary ($R^{2}=0.95$) and 94% for anxiety ($R^{2}=0.94$). The model was validated against empirical data, confirming high predictive accuracy.
